# Big Data, Big Opportunity: Using Normative Models to Bridge Cross‐Sectional and Longitudinal Insights

**DOI:** 10.1002/alz70856_103112

**Published:** 2025-12-26

**Authors:** Barbora Rehak Buckova

**Affiliations:** ^1^ Radboud University Medical Center, Nijmegen, Netherlands

## Abstract

The growth of large‐scale neuroimaging collaborations has opened new opportunities for analyzing data at an unprecedented scale, driving the development of methods that exploit data variation to deepen our understanding of both healthy population variation and disease‐specific patterns. Among these methods, normative modeling stands out as a powerful framework that relates individual neuroimaging data to population‐derived standards. This approach models an individual's neuroimaging phenotypes in relation to demographic and clinical variables within a large, healthy population, enabling subject‐level inferences about their position relative to the population.

While normative models excel in establishing robust population norms and identifying disease‐specific patterns, their potential for analyzing temporal dynamics has yet to be fully realized. The cross‐sectional nature of most datasets limits the ability to model subject‐level trajectories over time. Furthermore, existing methods for analyzing longitudinal data often fail to integrate insights from large‐scale cross‐sectional datasets, hindering the detection of early markers of neurodegeneration or subtle deviations from typical aging trajectories.

To address this limitation, we introduce an innovative normative modeling approach that leverages the heterogeneity in large cross‐sectional datasets and also incorporates longitudinal dynamics for subject‐specific inferences. This method builds upon pre‐trained normative models derived from over 58,000 individuals, offering scalability, flexibility, and applicability across studies with varying sample sizes. By comparing an individual's trajectory with the expected dynamics derived from population norms, our approach enables the detection of atypical changes that might otherwise go unnoticed. The result of this method is an individualized score, which quantifies whether a significant change has occurred in each individual over time, allowing for more precise detection of deviations from typical trajectories.

This technique holds particular promise for Alzheimer's disease research, where early detection and tracking of disease progression are critical. By integrating cross‐sectional and longitudinal data, our framework improves the ability to detect subtle changes over time, contributing to a more nuanced understanding of neurodegeneration. Furthermore, this approach is versatile and can be extended to other neurological and psychiatric conditions, providing a powerful tool for analyzing longitudinal neuroimaging data across a wide range of studies.

